# Analysis of Polycyclic Aromatic Hydrocarbons in *Heregovački pršut*—Traditionally Smoked Prosciutto

**DOI:** 10.3390/ijerph17145097

**Published:** 2020-07-15

**Authors:** Krešimir Mastanjević, Leona Puljić, Brankica Kartalović, Jozo Grbavac, Marija Jukić Grbavac, Helena Nadaždi, Kristina Habschied

**Affiliations:** 1 Faculty of Food Technology Osijek, Josip Juraj Strossmayer University of Osijek, F. Kuhača 20, 31000 Osijek, Croatia; Helena.Nadazdi@ptfos.hr; 2 The Faculty of Agriculture and Food Technology (APTF) of the University of Mostar, Biskupa Čule bb, 88000 Mostar, Bosnia and Herzegovina; leonapuljic224@gmail.com (L.P.); grbavac.jozo@gmail.com (J.G.); jgmarija@gmail.com (M.J.G.); 3 Scientific Veterinary Institute Novi Sad, Rumenački put 20, 21000 Novi Sad, Serbia; brankica@niv.ns.ac.rs

**Keywords:** Polycyclic aromatic hydrocarbons (PAHs), traditional smoking, *Hercegovački pršut*, prosciutto

## Abstract

*Hercegovački pršut* as a traditional dry-cured smoked ham (prosciutto) produced by using an open fire that can be potentially contaminated with polycyclic aromatic hydrocarbons (PAHs) and can pose a health risk for consumers. The aim of this research was to identify the types and concentrations of 16 PAHs in 34 samples of traditionally smoked prosciutto. Out of 16 investigated PAHs, identified in the EPA (Environmental Protection Agency) list of priority pollutants, 14 compounds were detected. Average levels of cancerogenic benzo[a]pyrene (BaP) and PAH4 (benzo[a]anthracene (BaA), benzo[b]fluoranthene (BbF), chrysene (Chry), and benzo[a]pyrene (BaP)) ranged from <LOQ (level of quantification) to 5.08 μg/kg and 0.45 μg/kg to 22.67 μg/kg. Two analyzed samples exceeded currently prescribed values according to the Bosnia and Herzegovina legislation for BaP concentrations and one sample for PAH4 content. PAH16 concentrations were on average between 2.92 μg/kg and 87.6 μg/kg. The highest PAH concentrations were found in samples from the Herzegovina-Neretva canton. The results of the research highlight the importance of standardizing smoking procedures and manufacturing practice, in the production of *Hercegovački pršut*, in order to reduce the PAH content.

## 1. Introduction

*Hercegovački pršut* is a traditional meat product of farms and is produced throughout Herzegovina. Today, traditional products, products with indications of originality, and products with indications of geographical origin are increasingly valued. Products whose special characteristics derive from the value of their ingredients, the method of production, and the processing and climate from which they come from get special praise on the market. Despite the fact that the production of *Hercegovački pršut* has a relatively long history, there is very little data on their production. Diversity in breed composition, method of breeding and fattening, feeding, final body weight of pigs (120–220 kg), primary processing of the pork leg, as well as technological process results in a large uneven appearance and quality of prosciutto. Traditionally, pigs fattened on their own farm are used for the production of *Hercegovački pršut*, and are mainly crossbreeds and hybrids of different white breeds of pigs, which are feed on barley, corn, rye, triticale, household leftovers, milk processing, beets, pumpkins, potatoes, various fruits, and by-products of the food industry. Fattening is carried out in a large percentage in an extensive manner and economic aspects are most often neglected [1]. *Hercegovački pršut* is a durable dried meat product from pork leg with bones, skin and subcutaneous fat, without pelvic bones, dry-salted mostly with sea salt with the possibility of adding other herbs, smoked by mild burning of hardwood beech (*Fagus* sp.), Oak (*Quercus* sp.), or hornbeam (*Carpinus* sp.), and subjected to a process of drying and ripening for 12 to 18 months.

However, due to the smoking procedure using open fire used in traditional production of *Hercegovački pršut,* special attention should be given to the smoking process because it may lead to the contamination with polycyclic aromatic hydrocarbons (PAHs). Incomplete wood combustion during the process of smoking meat leads to the formation of PAHs, a potential health hazard [2]. Smoking meat is the most common source of PAHs in food (type of wood, duration of smoking procedure, casing), however, environmental contamination also significantly contributes to the food contamination [3,4]. As such, meat products may be contaminated with PAHs related to the contamination of forage used in animal feeding.

**Figure 1:**
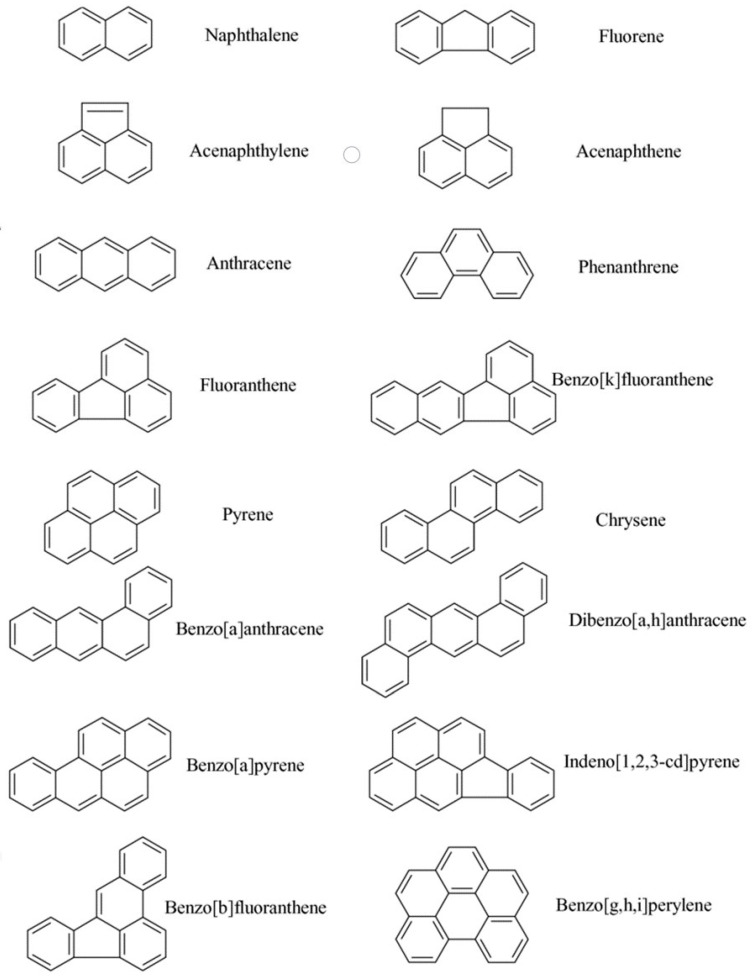
Structure formulas of the 16 priority PAHs (polycyclic aromatic hydrocarbons) according to US EPA.

Polycyclic aromatic hydrocarbons include a large group of toxicants, from the point of food safety and toxicity, with two or more aromatic rings made up of carbon and hydrogen atoms [5,6]. PAHs with four or less fused benzene rings are described as light and those that contain more than four rings are recognized as heavy PAHs. Heavy PAHs are known as more toxic and stable. Thus, the US Environmental Protection Agency (US EPA) defined 16 PAHs (naphthalene (Nap), acenaphthylene (Anl), acenaphthene (Ane), fluorene (Flu), phenanthrene (Phen), anthracene (Ant), fluoranthene (Flt), pyrene (Pyr), benz[a]anthracene (BaA), chrysene (Chry), benzo[b]fluoranthene (BbF), benzo[k]fluoranthene (BkF), benzo[a]pyrene (BaP), indeno [1–3-cd]pyrene (InP), dibenz[a,h]anthracene (DahA) and benzo[g,h,i]perylene (BghiP); PAH16) as priority environmental pollutants (Figure 1). EFSA (European Food Safety Agency) decided that from the point of view of food safety, it is necessary to perform the concentration of the following PAHs compounds: BaP and the sum of the concentrations of four PAHs: BaP, BaA, BbF, and Chry (PAH4) [7].

According to Bosnia and Herzegovina’s regulations on maximum levels for PAHs in food [8], the maximum permissible concentration of BaP in meat products is set at 2 µg/kg and the sum of PAH4 concentrations should not exceed 12 µg/kg. This in in agreement with EU regulation no. 835/2011 [9]. 

Recently, many researchers from EU countries [10,11,12,13,14] and some outside EU [15,16], have reported concerns about the potentially increased content of PAHs in various traditional meat products. Also, there are a few recent studies showing the influence of different production methods on PAH concentration in various meat products [17,18,19] 

Considering the lack of relevant information on PAH concentrations in dry-cured smoked prosciutto produced in Herzegovina, the aim of this research was to identify the types and concentrations of 16 PAHs in traditionally smoked prosciuttos.

## 2. Materials and Methods 

### 2.1. Sampling

For the purpose of this research, 34 dry-cured and -smoked *Hercegovački pršut* was sampled. The sampling was performed randomly over the year 2019 in the territory of Herzegovina—the Herzegovina-Neretva canton (municipalities: Mostar, Čitluk, Čapljina, Neum), the West Herzegovina canton (municipalities: Ljubuški, Grude, Široki Brijeg, Posušje), the Herzeg-Bosnia canton (municipalities: Livno, Tomislavgrad, Kupres), and the Trebinje region. All sampled dry-cured smoked prosciuttos were produced by representative homemade producers. Immediately after collection, the samples were transported to the laboratory, and kept in glass bottles till the beginning of analysis at a temperature below 4 °C. Raw prosciutto was processed to dry-cured prosciutto in the traditional way, characteristic for the region of Herzegovina. 

### 2.2. Production of Hercegovački Pršut

Primary technological processing involves the removal of the sacrum and pelvis, tail, and trotters, while the skin is left. In Herzegovina, the dry-salting process is started immediately after shaping the hams. Salting is done in traditional manner by vigorously rubbing an indefinite amount of salt (mainly sea salt) over all surfaces of the ham and leaving it lying with the medial side facing up. Salting is performed at a temperature of 0–6 °C and a relative humidity of more than 80%. After 7–10 days (depending on the weight of the prosciutto), it is necessary to rub the prosciuttos again with salt and lay it down for the next 7–10 days with the medial side facing down. At the end of the salting phase, the prosciutto pressing phase lasts for 10 to 15 days. The hams are pressed so that they are arranged in rows between the plates and the load. The main goal of this phase is to squeeze and shape the prosciutto. As in the salting phase, the temperature in the pressing phase must be 2–6 °C, and the relative humidity must be higher than 80%. Some producers, after the pressing phase, coat the prosciuttos with a certain amount of white garlic, red pepper, and pepper. Also, coating the raw prosciutto with these spices can be done after the smoking phase and before the ripening phase. Properly salted prosciuttos, washed and drained, are tied with twine or hung on a stainless-steel hook above the heel bump (*Tuber calcanei*) and transferred to the drier to even out the temperature before smoking. Even and gentle ventilation must be provided in the drier. After equalizing the temperature of salted and drained prosciuttos, with the temperature of the drier, the smoking phase follows, where the product is also dried. Smoking is done using cold smoke obtained by burning hardwood or sawdust of beech, oak, or hornbeam. Smoking is done in the classic way with an open fire. Combustion should be quiet (without flames) so it does not exceed the cold smoking temperatures. If the weather is humid, smoking is done continuously, and during dry days it is smoked only for a few hours during the day. The smoking phase in the traditional production of *Hercegovački pršut* lasts between 30 to 90 days. This phase is very important for the formation of the characteristic aroma of smoke. After the smoking and drying phase, the prosciutto is moved to ripen in a room with a stable microclimate, which has openings for air exchange (windows) due to the proper conduct of the technological process. All openings must be protected by a dense net that prevents the free entry of insects, rodents and other parasites. It is desirable for the temperature in the ripening rooms to not exceed 20 °C and the relative humidity to stay below 90%. In such microclimatic conditions, prosciutto loses moisture evenly and ripens properly. After 12 to 18 months from the start of salting, the prosciutto is ripe and ready for consumption. After the ripening stage was completed, the prosciutto was sampled for analysis, and all samples were vacuum packed, coded and stored in the dark at −30 °C. Each sample was analyzed in triplicate, therefore PAH values determined and presented in this paper represent the mean values of three parallel analyses. 

### 2.3. GC-MS (Gas Chromatography–Mass Spectrometry) Analysis

Chromatographic separation of 16 PAHs of interest, was performed by gas chromatography in combination with a mass detector (GC-MS) according to Mastanjević et al. [3] and Puljic et al. [20]. In short, standard solutions of PAHs were prepared with a PAH mix of 16 polycyclic aromatic hydrocarbons 500 ± 0.2 µg/mL (Ultra Scientific, North Kingstown, RI, USA). Method validation and calibration through a matrix blank sample was performed as well. Samples were prepared using the quick, easy, cheap, effective, rugged and safe preparation (QuEChERS) method. GC-MS parameters were adjusted as described in Supplementary Materials and reported by Mastanjević et al. [3]. All analyses were performed in triplicate.

### 2.4. Statistical Analysis

Experimental data were analyzed using an analysis of variance (ANOVA) and Fisher’s least significant difference (LSD), with significance defined at *p* < 0.05. Statistical analyses was carried out with Statistica 13.1. (TIBCO Software Inc., Palo Alto, CA, USA).

## 3. Results and Discussion

In order to improve safety and quality of traditionally produced meat products in Bosnia and Herzegovina, the control of PAH quantity in these products is relevant. As the smoking is done with open fire, it can lead to the formation of undesirable carcinogenic PAHs [21,22,23]. Also, PAH concentrations in traditionally produced *Hercegovački pršut* can be increased by the addition of spices, which oftentimes can be contaminated with PAHs [24]. In order to get information about the amounts and types of PAHs consumers are exposed to when they are consuming traditional *Hercegovački pršut*, the survey was conducted on samples produced in traditional matter.

Results of the research shown in Table 1 revealed the presence of 14 out of 16 investigated PAHs, identified in the EPA list of priority pollutants. 

The two substances Ane and Pyr were not detected. Examination results showing the domination of light PAHs was is in agreement with earlier studies on PAHs contaminations in smoked meat products [17,25,26,27]. Content of light PAHs determined in *Hercegovački pršut* involved Nap, Anl, Flu, Ant, Phen, Flt, and BaA. Nap, BaA and Ant were quantified in all 34 samples of *Hercegovački pršut*. Anl was quantified in 53%, Flu in 97.1%, Phen in 55.9%, and Flt in 47.1% of samples. Heavy PAHs determined in *Hercegovački pršut* samples were Chry (3.0%), BbF (55.9%), BkF (47.1%), BaP (41.2%), DahA (20.6%), BghiP (47.1%), and InP (50.0%).These results are in agreement with data on PAH profiles earlier reported by authors from surrounding countries, using similar technologies in the production of similar traditional smoked meat products [28,29,30].

PAH distribution in *Hercegovački pršut* samples according to the canton are shown in Table 1. The most abundant light PAH in all sample groups were Nap, which ranged from average 2.13 µg/kg in the West Herzegovina sample group to 1.14 µg/kgin the region of Trebinje. Anl ranged from 0.02 µg/kg in the region of Trebinje to 0.35 µg/kg in the Herzegovina-Neretva canton. Flu content was between 0.20 µg/kg in the region of Trebinje and 0.69 µg/kg in the West Herzegovina canton. Ant and Phen were between 0.95 µg/kg and 0.02 µg/kg in the Herzeg-Bosnia canton and 1.93 µg/kg and 0.73 µg/kg in the est Herzegovina canton and the Herzegovina-Neretva canton. Nap, Anl, Flu, Ant, and Phen content varied significantly (*p* < 0.05) within all sample groups. Flt and BaA content ranged between <LOQ (level of quantification) and 0.82 µg/kg in the region of Trebinje and 0.17 µg/kg and 3.04 µg/kg in the Herzegovina-Neretva canton. In all samples of *Hergovački pršut*, Ane and Pyr were not quantified. Heavy PAH concentrations were as follows: Chry and BbF which ranged from <LOQ in West Herzegovina, Herzeg-Bosnia, and the region of Trebinje to 0.46 µg/kg in the Herzegovina-Neretva canton with statistical significant difference (*p* < 0.05) between these three cantons and the Herzegovina-Neretva canton. BbF and Bkf concentrations were between <LOQ and 0.02 µg/kg in the Herzeg-Bosnia canton and 1.07 µg/kg and 0.68 µg/kg in the Herzegovina-Neretva canton and varied significantly (*p* < 0.05) within the cantons. BaP, InP, and Bghip ranged from <LOQ and 0.09 µg/kg in the Herzeg-Bosnia canton and 0.65 µg, 1.00 µg/kg, and 0.89 µg/kg, respectively, in the Herzegovina-Neretva canton. Daha concentrations were between <LOQ in the Herzeg-Bosnia canton and the region of Trebinje to 1.05 µg/kg in the Herzegovina-Neretva canton showing a statistically significant difference (*p* < 0.05) between the Herzeg-Bosnia canton and the region of Trebinje and all other sample groups. The highest concentration of all PAHs was detected in the Herzegovina-Neretva canton and the lowest was in the region of Trebinje. This might be explained by the fact that some producers are more aware of combustion conditions and they pay more attention to it. Also, some regions more often use different spices and herbs in traditional production of *Hercegovački pršut*, which can often be contaminated with PAHs [31]. Poljanec et al. [28] reported similar results for PAH concentrations in four different types of Croatian prosciuttos. On the other hand, Mastanjević et al. [17] reported significantly higher concentrations of PAHs in the dry-cured meat product *Slavonska šunka.* Also, Puljić et al. [20] reported higher levels of PAHs in the traditionally smoked dry-cured meat product *Hercegovačka pečenica.* This can be explained by the different smoking procedures in the traditional production of these products such as combustion conditions (distance from the fire, wood type, or fire height).

According to current Bosnia and Herzegovina’s legislation [8], the prescribed content of BaP in meat products is set at 2 µg/kg. As shown in Table 1 BaP concentrations ranged from <LOQ to 5.08 µg/kg (average 0.18 µg/kg). Two analyzed samples from the Herzegovina-Neretva canton exceed the prescribed values. Other analyzed samples had acceptable values for BaP content. Average levels of BaP were below those reported by other authors [17,30] and in agreement with those reported by Poljanec et al. [28] and Bogdanović et al. [29] in different types of Croatian prosciuttos. On the other hand, Mastanjević and al. [17] reported that BaP concentrations in traditional produced *Slavonska šunka* was below the LOQ. Content of PAH4 and PAH16 in *Hercegovački pršut* are presented in Table 1.

One sample analyzed in this research, from the Herzegovina-Neretva canton with content of 22.67 µg/kg for PAH4, exceed the prescribed values of 12 µg/kg. Average content of PAH4 in all 34 samples of *Hercegovački pršut* were 2.32 µg/kg showing significant variation (*p* < 0.05) between all cantons of production. Distribution of PAH4 according to the production place (canton) in *Hercegovački pršut* is also shown in Table 1. The highest concentrations of PAH4 were found in the Herzegovina-Neretva canton that showed significantly higher PAH4 values compared to other cantons. This can be explained by the non-standardized smoking practices in the traditional production of these prosciuttos. PAH16 concentrations were on average 10.10 µg/kg, while the lowest average concentrations were in the region of Trebinje (5.21 µg/kg) and the highest were detected in the Herzegovina-Neretva canton (19.60 µg/kg). Still, collected data from this research showed that the samples with higher PAH4 and BaP concentrations also had higher PAH16 concentrations. The levels of PAH4 and PAH16 detected in this research are above those reported by Ciecierska et al. [32] for traditionally smoked prosciutto. Djinovic et al. [25] and Kartalović et al. [30] also reported higher concentrations of PAH4 in traditionally produced pork prosciutto from Serbia. Similar results were observed in research reported by Poljanec et al. [28] and Bogdanović [29] for different types of traditionally produced Croatian dry-cured and smoked prosciutto.

## 4. Conclusions

The results of research revealed presence of 14 out of 16 investigated PAHs, identified in the EPA list of priority pollutants. Content of light PAHs involved Nap, Anl, Flu, Ant, Phen, Flt, and BaA. Determined heavy PAHS were Chry, BbF, BkF, BaP, DahA, BghiP, and InP. Two analyzed samples exceed currently prescribed values according to BIH (Bosnia and Herzegovina) legislation for BaP concentrations and one sample for PAH4 content. Although the maximum levels for PAH16 concentrations in meat products are not currently prescribed in EU or BIH legislation, there is a certain correlation between PAH16 content and PAH4 and BaP concentrations. This research showed that samples with higher PAH4 and BaP content also show higher PAH16 content. The highest PAH content are found in samples from the Herzegovina-Neretva canton. Results highlight the importance of standardizing smoking procedures and good manufacturing practice (in terms of used wood, minimal height during smoking with open fire, ventilation, and used herbs) in the production of traditional *Hercegovački pršut* in order to reduce the PAH content.

## Figures and Tables

**Table 1:** PAH (polycyclic aromatic hydrocarbons) contents (µg/kg) in *Hercegovački pršut*.

PAH	Herzegovina-Neretva Canton (*N* = 12)	West Herzegovina Canton (*N* = 10)	Herzeg-Bosnia Canton (*N* = 7)	Trebinje Region (*N* = 5)	Total (*N* = 34)
**Nap**					
<LOQ (%)	0	0	0	0	0
Min–Max μg/kg	0.01–12.99	0.01–10.09	0.72–2.66	0.51–1.97	0.01–12.99
Average ± St. Dev μg/kg	1.84 ^b^ ± 3.35	2.13 ^a^ ± 2.63	1.42 ^d^ ± 0.69	1.14 ^e^ ± 0.41	1.63 ^c^ ± 2.47
**Anl**					
<LOQ (%)	4(33.3)	3(30.0)	6(85.7)	4(80.0)	16(47.0)
Min–Max μg/kg	<LOQ–2.32	<LOQ–1.63	<LOQ–0.19	<LOQ–0.10	<LOQ–2.32
Average ± St. Dev μg/kg	0.35 ^a^ ± 0.62	0.30 ^b^ ± 0.49	0.02 ^d^ ± 0.06	0.02 ^e^ ± 0.04	0.17 ^c^ ± 0.48
**Ane**					
<LOQ (%)	12(100.0)	10(100.0)	7(100.0)	5(100.0)	34(100.0)
Min–Max μg/kg	<LOQ	<LOQ	<LOQ	<LOQ	<LOQ
Average ± St. Dev μg/kg	<LOQ	<LOQ	<LOQ	<LOQ	<LOQ
**Flu**					
<LOQ (%)	1(8.3)	0	0	0	1(2.9)
Min–Max μg/kg	<LOQ–1.07	0.19–2.45	0.11–0.50	0.11–0.34	<LOQ–2.45
Average ± St. Dev μg/kg	0.67 ^b^ ± 0.29	0.69 ^a^ ± 0.63	0.27 ^d^ ± 0.10	0.20 ^e^ ± 0.07	0.45 ^c^ ± 0.43
**Ant**					
<LOQ (%)	0	0	0	0	0
Min–Max μg/kg	0.86–2.86	0.54–5.06	0.50–2.04	0.67–1.37	0.5–5.06
Average ± St. Dev μg/kg	1.70 ^b^ ± 0.52	1.93 ^a^ ± 1.55	0.95 ^e^ ± 0.44	0.97 ^d^ ± 0.24	1.39 ^c^ ± 0.99
**Phen**					
<LOQ (%)	1(8.3)	5(50.0)	6(85.7)	4(80.0)	15(44.1)
Min–Max μg/kg	<LOQ–5.97	<LOQ–1.25	<LOQ–0.14	<LOQ–0.14	<LOQ–5.97
Average ± St. Dev μg/kg	0.73 ^a^ ± 1.22	0.25 ^b^ ± 0.40	0.02 ^d^ ± 0.05	0.02 ^c^ ± 0.06	0.26 ^b^ ± 0.82
**Flt**					
<LOQ (%)	3(25.0)	5(50.0)	6(85.7)	5(100.0)	18(52.9)
Min–Max μg/kg	<LOQ–0.50	<LOQ–0.44	<LOQ–0.20	<LOQ	<LOQ–050
Average ± St. Dev μg/kg	0.17 ^a^ ± 0.14	0.14 ^b^ ± 0.15	0.03 ^d^ ± 0.07	<LOQ	0.08 ^c^ ± 0.14
**Pyr**					
<LOQ (%)	12(100)	10(100.0)	7(100.0)	5(100.0)	34(100)
Min–Max μg/kg	<LOQ	<LOQ	<LOQ	<LOQ	<LOQ
Average ± St. Dev μg/kg	<LOQ	<LOQ	<LOQ	<LOQ	<LOQ
**BaA**					
<LOQ (%)	0	0	0	0	0
Min–Max μg/kg	0.93–6.85	0.23–4.37	0.72–1.96	0.29–2.02	0.23–6.85
Average ± St. Dev μg/kg	3.04 ^a^ ± 1.46	1.52 ^c^ ± 1.24	1.24 ^d^ ± 0.45	0.82 ^e^ ± 0.61	1.66 ^b^ ± 1.43
**Chry**					
<LOQ (%)	11(91.6)	10(100.0)	7(100.0)	5(100.0)	33(97.0)
Min–Max μg/kg	<LOQ–6.07	<LOQ	<LOQ	<LOQ	<LOQ–6.07
Average ± St. Dev μg/kg	0.46 ^a^ ± 1.58	<LOQ	<LOQ	<LOQ	0.11 ^b^ ± 0.96
**BbF**					
<LOQ (%)	3(25.0)	4(40.0)	7(100.0)	2(40.0)	15(44.1)
Min–Max μg/kg	<LOQ–5.10	<LOQ–0.67	<LOQ	<LOQ–0.24	<LOQ–5.10
Average ± St. Dev μg/kg	1.07 ^a^ ± 1.84	0.23 ^c^ ± 0.25	<LOQ	0.11 ^d^ ± 0.10	0.35 ^b^ ± 1.2
**BkF**					
<LOQ (%)	4(33.3)	5(50.0)	6(85.7)	4(80.0)	18(52.9)
Min–Max μg/kg	<LOQ–7.91	<LOQ–0.16	<LOQ–0.14	<LOQ–0.18	<LOQ–7.91
Average ± St. Dev μg/kg	0.68 ^a^ ± 1.98	0.07 ^c^ ± 0.07	0.02 ^e^ ± 0.05	0.03 ^d^ ± 0.07	0.20 ^b^ ± 1.21
**BaP**					
<LOQ (%)	4(33.3)	7(70.0)	7(100.0)	3(60.0)	20(58.8)
Min–Max μg/kg	<LOQ–5.08	<LOQ–0.18	<LOQ	<LOQ–0.14	<LOQ–5.08
Average ± St. Dev μg/kg	0.65 ^a^ ± 1.38	0.04 ^c^ ± 0.07	<LOQ	0.04 ^c^ ± 0.06	0.18 ^b^ ± 0.87
**DahA**					
<LOQ (%)	8(66.6)	8(80.0)	7(100.0)	5(100.0)	27(79.4)
Min–Max μg/kg	<LOQ–12.83	<LOQ–1.24	<LOQ	<LOQ	<LOQ–12.83
Average ± St. Dev μg/kg	1.05 ^a^ ± 3.15	0.16 ^c^ ± 0.38	<LOQ	<LOQ	0.31 ^b^ ± 1.95
**BghiP**					
<LOQ (%)	7(58.3)	4(40.0)	6(85.7)	2(40.0)	18(52.9)
Min–Max μg/kg	<LOQ–7.43	<LOQ–1.44	<LOQ–0.65	<LOQ–1.10	<LOQ–7.43
Average ± St. Dev μg/kg	0.89 ^a^ ± 2.01	0.41 ^c^ ± 0.44	0.09 ^d^ ± 0.24	0.45 ^bc^ ± 0.47	0.47 ^b^ ± 1.27
**InP**					
<LOQ (%)	6(50.0)	5(50.0)	7(100.0)	2(40.0)	17(50.0)
Min–Max μg/kg	<LOQ–10.02	<LOQ–2.41	<LOQ	<LOQ–0.87	<LOQ–10.02
Average ± St. Dev μg/kg	1.00 ^a^ ± 2.46	0.46 ^b^ ± 0.70	<LOQ	0.37 ^c^ ± 0.37	0.46 ^b^ ± 1.56
**PAH4**					
<1 (%)	1(8.33)	1(10.0)	3(42.8)	3(60.0)	7(20.5)
Min–Max μg/kg	0.95–22.67	0.45–4.67	0.72–1.96	0.52–2.15	0.45–22.67
Average ± St. Dev μg/kg	5.24 ^a^ ± 5.48	1.81 ^c^ ± 1.15	1.24 ^d^ ± 0.45	0.99 ^e ±^ 0.56	2.32 ^b^ ± 3.81
**PAH16**					
<1 (%)	0	0	0	0	0
Min–Max μg/kg	6.71–87.65	3.74–23.28	2.92–9.83	3.17–7.95	2.92–87.6
Average ± St. Dev μg/kg	19.60 ^a^ ± 20.60	10.21 ^b^ ± 5.44	5.35 ^d^ ± 1.86	5.21 ^e^ ± 1.43	10.10 ^c^ ± 14.05

Values in the same row with different superscripts (a–e) are significantly different (*p* < 0.05); naphthalene (Nap), acenaphthylene (Anl), acenaphthene (Ane), fluorene (Flu), phenanthrene (Phen), anthracene (Ant), fluoranthene (Flt), pyrene (Pyr), benz[a]anthracene (BaA), chrysene (Chry), benzo[b]fluoranthene (BbF), benzo[k]fluoranthene (BkF), benzo[a]pyrene (BaP), indeno [1–3-cd]pyrene (InP), dibenz[a,h]anthracene (DahA) and benzo[g,h,i]perylene (BghiP); PAH4 ∑ BaP, BaA, BbF, and Chry; PAH16 ∑ Nap, Anl, Ane, Flu, Ant, Phen, Flt, Pyr, BaA, Chry, BbF, BkF, BaP, DahA, BghiP and InP; LOQ - limit of quantification.

## Supplementary Materials

The following are available online at https://www.mdpi.com/1660-4601/17/14/5097/s1, Table S1: The average values for precision, reproducibility, accuracy, linearity, LOQ and LOD for PAH method validation.
