# Supplementary material for: Analysis of Polycyclic Aromatic Hydrocarbons in Heregovački pršut—Traditionally Smoked Prosciutto
Source: Int J Environ Res Public Health. 2020 Jul 15;17(14):5097. doi: 10.3390/ijerph17145097 (PMC7400239; doi:10.3390/ijerph17145097)
Supplement: Supplementary file 1 [file ijerph-17-05097-s001.pdf]

Standard solutions of polycyclic aromatic hydrocarbons (PAHs) were prepared with PAH mix of 16 polycyclic aromatic hydrocarbons (Ultra Scientific, North Kingstown, USA), 500 ± 0.2 µg/ml. In order to eliminate the matrix influence, a calibration through matrix blank sample was performed as well. The retention times of the peaks and target ions obtained from the standard solution of PAHs served as a base point for PAH determination in samples.

Samples were prepared using multiresidue preparations that ensure quick, easy, cheap, effective, rugged and safe preparation (QuEChERS), adapted from the Association of Analytical Communities (AOAC) Official method 2007.01). In short, the method included extraction using acetonitrile (ACN, Sigma-Aldrich, St. Louis, Missouri, USA) in the presence of anhydrous magnesium sulphate (MgSO4; Merck, Darmstadt, Germany) and anhydrous sodium acetate (CH_3COONa; Merck, Darmstadt, Germany). Three g of sample were transferred into the centrifuge tube where a mixture of 3 ml of acetonitrile and 3 ml of water was added. After the intensive stirring on a vortex for one min, 3 g of anhydrous magnesium sulphate and 1 g of anhydrous sodium acetate were added. The sample was then centrifuged for 5 min at 3000 rpm and 1 ml of upper layer of the acetonitrile extract got transferred into the 5 ml tube, along with 150 mg of anhydrous magnesium sulphate, 100 mg of Primary and Secondary Amine (PSA) (Merck, Darmstadt, Germany) and 50 mg of C18 (Merck, Darmstadt, Germany). The content was again centrifuged for 5 min at 3000 rpm, ensuring the clear and pure extract. A 0.5 ml of the extract was then subjected to evaporation under nitrogen gas and reconstituted with hexane, resulting in a sample ready for the analysis on GC-MS (Agilent 7890B/5977A, Santa Clara, CA, USA).

In short, a DB-5MS column (30 m × 0.25 µm × 0.25 mm) (Agilent J&W, Santa Clara, CA, USA) was used to separate PAH molecules. Sample volume of 4 µl (splitless mode) was injected at the constant pressure of 11.36 psi and flow through the column of the carrier gas of 1.2 mL/min. The target and qualifier abundances were determined by injection mixture of PAHs standards under the same chromatographic conditions. A full scan with the mass/charge ratio ranging from 60 to 500 m/z was employed. In order to minimize the matrix effect, standard solutions were prepared in blank matrix extracts. With the aim of obtaining more reliable results, further PAHs quantification is performed in SIM mode and the obtained data were processed using Mass Hunter Software. The analysis of the method performance is performed in calibration range from 0.005 to 0.1 mg/kg. The standard solution of PAHs mix served as a base for quantification using matrix calibration curves. The coefficients of determination (r^2) for PAHs standard calibration plots were above 0.99.

Agilent 7890B/5977A MSD, gas - mass chromatography was used for the analysis. The GC operating conditions were as following: fused silica column [30m x 0.25µm film of HP-5M (thickness)]; injection temperature was set at 280 °C using splitless mode and volume injected was 4 µl. The column temperature was programmed as following: hold at 50 °C for 0.4min; 50-195 °C at 25 °C/min, hold 1.5 min; 195-265 at 8 °C/min and maintained at 315 °C for 1.25 minutes on 20 °C/min, MSD temperature was 280 °C. The verification of peaks was done comparing retention times and target ions. Procedural blank and solvent blanks were analysed and quantified, but no PAHs were found in these blanks.

PAHs determination method was modified according to the accredited method ISO 17025. Validation referred to the determination of precision, reproducibility, accuracy, linearity, LOQ (limit of quantification), LOD (limit of detection) and uncertainty. The method precision was evaluated by repeatability using smoked meet spiked with PAH concentrations and analysed in triplicate (50.0 µg/kg, n = 20).

Accuracy was calculated using recovery values. Linearity of the detector was tested (5 to 500 µg/kg) and showed as satisfactory for all ranges. The LOD (0.29 to 0.5 µg/ kg) and the LOQ (1.05 to 2 µg/kg) (Table S1) values appeared somewhat higher than the levels set by the European Commission Regulation No.836/2011.

Table S1. The average values for precision, reproducibility, accuracy, linearity, LOQ and LOD for PAH method validation.

PAHs	Precision (%)	Reproducibility (%)	Accuracy (%)	Linearity (r^2)[a]	LOQ µg/kg	LOD µg /kg
Nap	11.3	6.33	95.0	0.99	1.20	0.30
Anl	7.91	7.82	99.0	0.99	1.30	0.29
Ane	8.52	8.32	99.3	0.99	1.05	0.32
Flu	2.82	10.2	100	0.99	1.11	0.30
Ant	3.53	3.73	98.7	0.99	1.10	0.30
Phen	4.31	11.4	85.9	0.99	1.18	0.35
Flt	3.61	3.72	95.3	0.99	1.15	0.30
BaA	9.44	8.6	89.7	0.99	1.30	0.37
Pyr	4.74	6.91	91.1	0.99	1.21	0.32
Chry	5.33	8.20	92.5	0.99	1.13	0.34
BbF	8.52	14.3	86.4	0.99	1.30	0.36
BkF	3.51	3.32	94.3	0.99	1.21	0.32
BaP	3.23	3.81	96.8	0.99	2.00	0..53
DahA	8.72	11.3	91.2	0.99	1.99	0.51
BghiP	9.71	11.3	81.5	0.99	1.90	0.45
InP	9.51	10.3	85.3	0.99	1.91	0.53
min	2.82	3.32	81.5	0.99	1.05	0.30
max	11.3	14.3	100	0.99	1.81	0.50

(r^2)[a] – correlation coefficient. Nap – naphthalene; Anl – acenaphthylene; Ane – acenaphtene; Flu – fluorene; Ant – anthracene Phen – phenanthrene; ; Flt – fluoranthene; BaA –benzo[a]anthracene Pyr – pyrene; ; Chry – chrysene; BbF – benzo[b]fluoranthene; BkF – benzo[k]fluoranthene; BaP – benzo[a]pyrene; DahA – dibenzo[a;h]anthracene; BghiP – benzo[g;h;i]-perylene; InP – indeno[1;2;3-cd]pyrene; LOD - limit of detection; LOQ - limit of quantification.
